# Effect of dry needling and instrumental myofascial release on masticatory, facial, and cervical muscles of patients with temporomandibular disorders of muscular origin

**DOI:** 10.4317/jced.60312

**Published:** 2023-05-01

**Authors:** Paulo-Roberto-Quiudini Junior, Selma Siéssere, Edneia-Corrêa de Mello, Sergio Rodrigues, Isabela Regalo, Ligia-Maria-Napolitano Gonçalves, Veridiana-Wanshi Arnoni, Marcelo Palinkas, Simone Regalo

**Affiliations:** 1 DDS. Department of Basic and Oral Biology, Ribeirão Preto School of Dentistry, University of São Paulo, Brazil; 2 DDS, PhD, Professor. Department of Basic and Oral Biology, Ribeirão Preto School of Dentistry and National Institute and Technology - Translational Medicine (INCT.TM), University of São Paulo, Brazil

## Abstract

**Background:**

To evaluate the effectiveness of dry needling (DN) and instrumental myofascial release (IMR) therapies in the cervico-cranio-mandibular system through pain, bite force, and distribution of occlusal contacts in patients with muscular temporomandibular disorders.

**Material and Methods:**

Thirty patients were divided into treatment groups: DN (n=15) and IMR (n=15). Therapeutic efficacy regarding pain perception and tolerance of masticatory, facial, and cervical muscles, bite force, and distribution of occlusal contacts were analyzed in this observational longitudinal clinical study pre/post-intervention and pre/post one month of therapeutic intervention. The data were tabulated and statistically analyzed (repeated measures and Bonferroni post-hoc test, *p*<0.05).

**Results:**

There was a statistically significant difference in pain between the groups in the comparison of pre- and post-intervention with effect on time versus intervention in the head and neck. Pain perception and tolerance showed a statistical effect of time on the temporal, suboccipital, sternocleidomastoid, mental (right and left), right masseter, and left trapezius muscles. There was a statistically significant effect of the intervention on the mentalis, supraorbital, and infraorbital (right and left) muscles. There was a statistically significant effect of the interaction on the upper masseter (right and left), anterior temporal (left), suboccipital, sternocleidomastoid, and mentalis (left) muscles. There was an increase in post-intervention molar bite force in the groups, with a statistical effect on time versus intervention in the right and left regions. Contact of occlusal forces at the maxilla/mandible interface showed a difference between the mean times on teeth 26–36 after versus 1 month after the intervention.

**Conclusions:**

The two therapeutic techniques are viable for the treatment of muscular temporomandibular disorders; however, IMR proved to be more effective immediately after the intervention and after one month.

** Key words:**Temporomandibular disorders, pain, masticatory muscles, facial muscles, cervical muscles, dry needling, instrumental myofascial release.

## Introduction

The temporomandibular joint is considered a complex anatomical structure when observing biomechanics. It has a pair of interdependent condyles, in which harmonic work is needed between the sides to carry out the movements, and any modification will exceed the physiological tolerance of these structures (1).

Among the functional alterations that compromise the temporomandibular joint, temporomandibular disorder (TMD) stands out. It encompasses a series of clinical problems involving the masticatory muscles, temporomandibular joint, and associated structures, and is characterized by the presence of pain, joint sounds, and irregular or limited mandibular function (2). It is difficult to diagnose and is classified as muscular, joint, or mixed (3).

Temporomandibular dysfunction can involve the entire stomatognathic system, which is an important system of the human body that allows the processing and transport of food from the oral cavity to the internal part of the body in a safe way, as well as assisting in the processes of phonation, breathing, and swallowing (4).

This musculoskeletal chronic pathological clinical condition is associated with myofascial pain, which also involves the function of the head and neck and becomes a syndrome with the presence of myofascial trigger points in the involved muscles, mainly the masticatory muscles (5). It is commonly found in females and more than 85% of patients are referred to clinics specializing in the control and management of painful symptoms (6). The therapeutic approach to deactivating the trigger points for muscle pain is discussed in the literature, which is to identify the most effective invasive or non-invasive technique for removing pain (7).

Functional evaluation methods are also fundamental for the analysis of the muscles of the cervico-cranio-mandibular system, as they allow the study of the muscular system after the application of therapeutic techniques. These provide important results on morphology and levels of functional activity and contribute to patient recovery (8).

Therefore, this study aimed to evaluate the effectiveness of DN and IMR therapies in the cervico-cranio-mandibular system through pain, bite force, and distribution of occlusal contacts in patients with muscular TMD. The null hypothesis was that the two interventions do not improve the pain symptoms and the performance of the cervico-cranio-mandibular system in patients with muscular TMD.

## Material and Methods

This observational longitudinal clinical study was approved by the ethics committee (process # 23555119.3.0000.5419). All the participants provided written informed consent.

-Sample

The a priori sample calculation performed using the G* Power 3.1.9.2 software (Franz Faul, Kiel University, Kiel, Germany) considered the level of α = 0.05, test power of 0.95, and an effect size of 1.56 for the main result of the pain threshold on palpation of the study by Blasco-Bonora and Martín- Pintado-Zugasti (9) (mean [SD] masseter muscle: pre-intervention, 1.54 [0.27] and one week after intervention, 2.10 [0.4]).

The minimum sample size obtained for this study was six patients; however, depending on the type of treatment applied, 30 patients aged between 18 and 60 years of both sexes, were divided into two treatment groups: DN (GDN, n=15) and IMR (GLMI, n=15). There was a pairing between the participants by sex, age, weight, and height (Table 1).

**Table 1:**
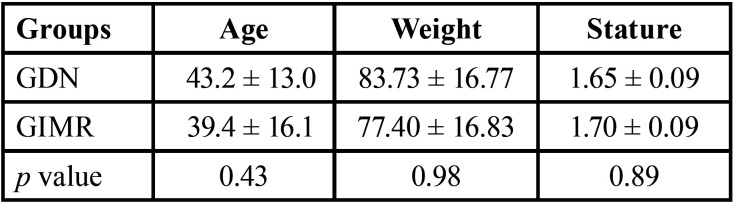
Differences in characteristics (mean ± standard deviation) between the dry needling (GDN) and instrumental myofascial release (GIMR). Significant difference, Student’s t-test (*p* < 0.05).

Information regarding personal data, medical and dental history, presence of systemic diseases, parafunctional habits, and possible signs and symptoms of TMD was obtained. Therapeutic efficacy regarding pain perception and tolerance of masticatory, facial, and cervical muscles, bite force, and distribution of occlusal contacts were analyzed pre/post-intervention and pre/post one month of therapeutic intervention. The treatment was applied on two occasions separated by one month.

Exclusion criteria were as follows: absence of cognitive system integrity; having a poorly adapted fixed prosthesis, a removable prosthesis, or a complete prosthesis; presence of periodontal disease (mild, moderate, or severe tooth mobility) or restorations at risk of fractures; physical or mental indisposition present at the time of the examination; a clinical history of systemic diseases that require chronic medication, such as neurological and psychiatric disorders, as well as users of medications that could interfere with muscle activity; absence of all teeth; orthodontic treatment; speech therapy; physiotherapy or otolaryngology (previous or current).

Inclusion criteria were as follows: age between 18 and 60 years, presence of TMD of muscular origin (Diagnostic Criteria for Temporomandibular Disorders - RDC/TMD), presence of pain and trigger points in the masseter muscles for more than three months with a score greater than three on the visual analog pain scale.

Participants were confirmed with muscle TMD by digital palpation. To be included in the research, they demonstrated one or more muscle diagnoses for TMD, thus being classified in one or more of the subgroups of diagnoses of Axis I of the RDC/TMD: myofascial pain without limitation of mouth opening and myofascial pain with limitation of mouth opening (10).

The DN technique was performed by needling the myofascial trigger points using short needles with a caliber and length of 0.20x13mm. The disposable and individual needles were placed in a plastic guide tube. The technical procedure consisted of inserting and deepening the needle by approximately 1 cm, and subsequently, the slow movements of insertion and partial removal of the needle in the region began. At each withdrawal, the insertion angle was modified in a circular manner, covering the entire region of the myofascial trigger points (11).

The IMR technique was performed with a modified instrument called Gancho QuiuTech (QuiuTech, Catanduva, Brazil), which is composed of two stainless steel rods with active tips responsible for instrumentation, and an aluminum cable in which the rods were joined with the function handle developed within the anatomical and technical concepts, providing a dynamic instrumentation technique. The instrument was applied for approximately 20 s in a direction parallel to the muscle fibers treated with the instrument at an angle of 45°. The instrument was immediately manipulated into the muscles in a direction perpendicular to the muscle fibers with the instrument at a 45° angle for an additional 20 s, resulting in a total treatment time of approximately 40 s (12).

-Pain intensity analysis

The visual analog scale (VAS) was used to indicate the intensities of pain before and immediately after performing the therapies. The scale consisted of a horizontal or vertical line of 100 millimeters (mm) or 10 centimeters (cm) with the ends marked as “no pain” and “maximum pain.” The patient registered a point on the line or between the extremes, and the researcher took measurements. Each VAS was completed on a separate piece of paper to avoid bias from the previous records. The patient was marked with a vertical line cutting the scale, where he believed his pain was located at the time of the examination.

-Maximum molar bite force analysis

The maximum molar bite force was recorded using a digital dynamometer (model IDDK; Kratos - Equipamentos Industriais Ltda, Cotia, São Paulo, Brazil), with a capacity of up to 980,665 Newtons, adapted for oral conditions. This equipment was provided by two rods that contained Teflon disks at the ends on which the bite force was applied (13).

At the time of bite force analysis, the patients remained seated in a position corresponding to the meato-orbital plane, parallel to the ground, and relaxed with the palms of the hands resting on the thighs. The bite struts were disinfected with alcohol and protected using disposable latex fingers (Wariper, São Paulo, Brazil) to avoid cross-contamination.

The patients received instructions and performed tests by squeezing the rods of the equipment in the region of the first permanent molars on both sides before the records were obtained to ensure the reliability of the procedure. The patients were then asked to bite the rods with maximum effort to collect the maximum force. Six measurements were performed, with a 2-min interval between them, with alternating sides; that is, there were three measurements on the right side and three on the left side with alternating measurements per side. The highest strength measurements obtained on each side were adopted.

-Evaluation of force distribution and occlusal analysis

The evaluation of the occlusal force distribution was performed using the Occlusense digital equipment (Dr. Jean Bausch GmbH & Co. KG, Koln, Germany) with a sensor that transmitted data via a Wi-Fi connection to the iPad application. The Occlusense tag could record up to 0.056 frames/s. The outer surfaces of the sensor were coated with an articulated red paint that marked the teeth simultaneously with the same time as the digital recording of the data.

Occlusense has 256 strength levels with a four-color-coded scheme (green/yellow/orange/red). This color gradient demonstrated the distribution of the masticatory force in the evaluated area and the difference in pressure between the pressure points through the height of the pixels. Pixel color and height indicate the differences in contact with adjacent contacts. This equipment made it possible to dynamically record the relative strength of the arches and the distribution of dental contacts over time (14).

To perform the procedure, the patients were instructed to remain seated in a chair without head support, with their feet flat on the floor. The sensor was positioned between the dental arches and was adjusted to the oral cavity. The patients were instructed to press the sensor on maximum dental intercuspation and apply the maximum force for six seconds, which was constant from the beginning to the end of the procedure. Two recordings were performed with an interval between the resting muscles. 

The system provides a graph with continuous recording. After performing the examination, the occlusal force distribution between the left and right sides and occlusal force distribution on teeth 16, 26, 36, and 46 were recorded. The occlusal analysis was performed using the OccluSense-iPad-App.

-Pressure pain threshold analysis

Pressure pain threshold was measured in Kgf/cm2 using an algometer (model DDK, Kratos®, Cotia, São Paulo. Brazil). The points chosen to measure the pressure pain threshold were the masseter, temporal, suboccipital, sternocleidomastoid, trapezius, supraorbital, infraorbital, and mentalis muscles.

The patient was guided by the examiner in relation to pressure in order to advise him to activate the device when the increasing stimulus exerted on his face ceased to be a pressure and became painful. The patient was instructed not to endure the pain but to register the moment when the pain started.

Prior to muscle assessment, a measurement was performed on each patient’s wrist to familiarize them with the methodology (15). The pressure pain threshold was measured with the algometer perpendicular to the point to be examined, exerting increasing and constant pressure of approximately 0.5 kg/cm2/s at each pre-determined point, until the patient reported symptoms of pain. At that moment, the pressure was no longer applied, and the algometer recorded the value.

-Statistical analysis

Repeated-measures analysis of variance (ANOVA) was performed to calculate the main effects of time (pre- versus post- versus pre-1 month versus post-1 month), group (GDN versus GLMI), and interaction (time versus intervention). Sphericity was tested using the Mauchly test, and homogeneity of variance was tested using Levene’s test. If sphericity was violated, the Greenhouse-Geisser correction was used. In the case of a significant effect of time and intervention, multiple comparison tests were used. Cohen’s coefficient was used to estimate the effect size (η2), interpreted as small (η2 = 0.2), medium (η2 = 0.5), or large (η2 = 0.8). A significance level of 5% was considered statistically significant. All analyses were performed using the Statistical Package for Social Sciences (version 20.0, SPSS Inc., Chicago, IL, USA).

## Results

Table 2- Table 2 cont.-1 shows the study variables, time, intervention, and interaction between DN (GDN) and IMR (GIMR) techniques. A reduction in the mean values after the intervention was observed for GDN and GIMR, with statistical effects of time and interaction (time versus intervention) in the head and neck region. An increase in the mean values of post-intervention bite force was observed for GDN and GIMR, with a statistical effect of time and interaction (time versus intervention) for the right and left molar regions. There were no statistical effects of time, intervention, or interaction (time versus intervention) on the maxilla/mandible interface between GDN and GIMR. Bonferroni’s multiple comparison tests revealed a statistical difference between the time means for teeth 26–36 after one month (*p*=0.018). A statistical effect of time was observed for the upper, middle, and lower right masseter; middle left masseter; anterior, middle, and posterior right and left temporal; right and left suboccipital; right and left sternocleidomastoid; left trapezius; and right and left mental muscles. There was a statistical effect of the intervention on the right and left supraorbital, right and left infraorbital, and right and left mental muscles. There was a statistical effect of the interaction between the right and upper left masseter, anterior left temporal, left suboccipital, left sternocleidomastoid, and left mental muscles.

**Table 2:**
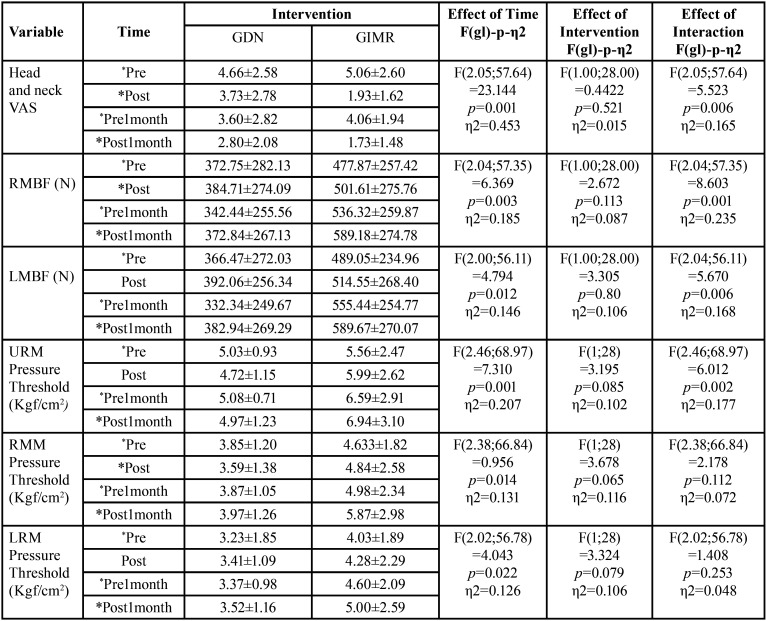
Mean, standard deviation (±), degree of freedom (df), effect size (η2), and p value (< 0.05) of the significant study variables, time, intervention and interaction of Dry Needling (GDN) and Instrumental Myofascial Release (GIMR) techniques.

**Table 2 cont.**
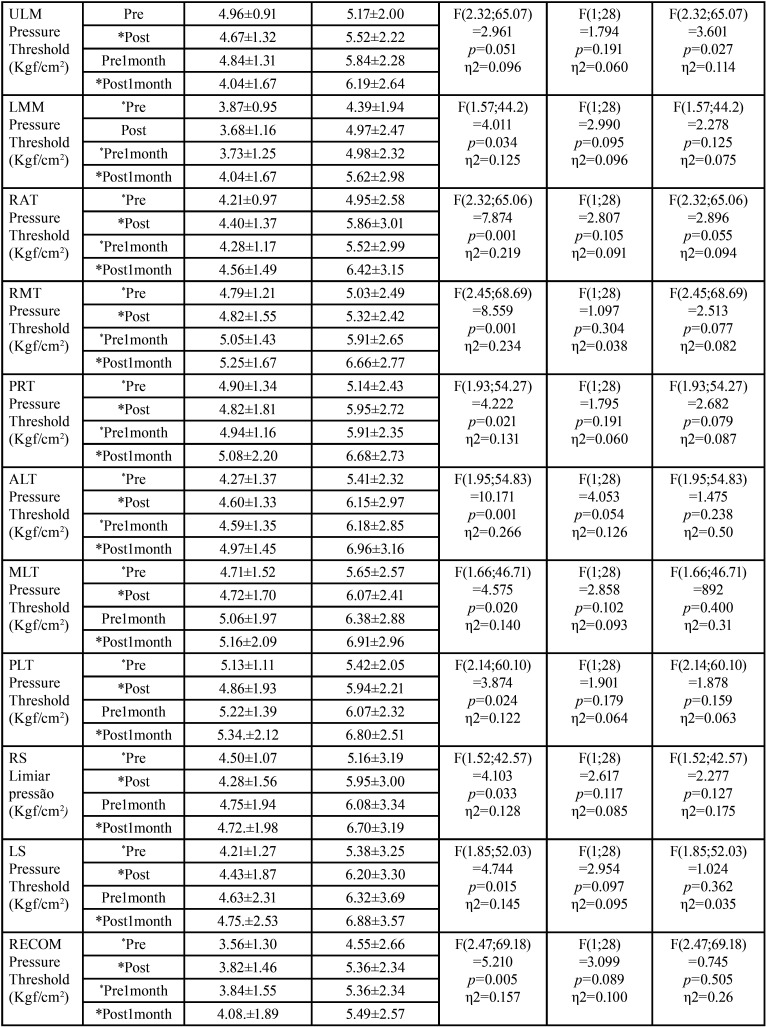
Mean, standard deviation (±), degree of freedom (df), effect size (η2), and p value (< 0.05) of the significant study variables, time, intervention and interaction of Dry Needling (GDN) and Instrumental Myofascial Release (GIMR) techniques.

**Table 2 cont.-1:**
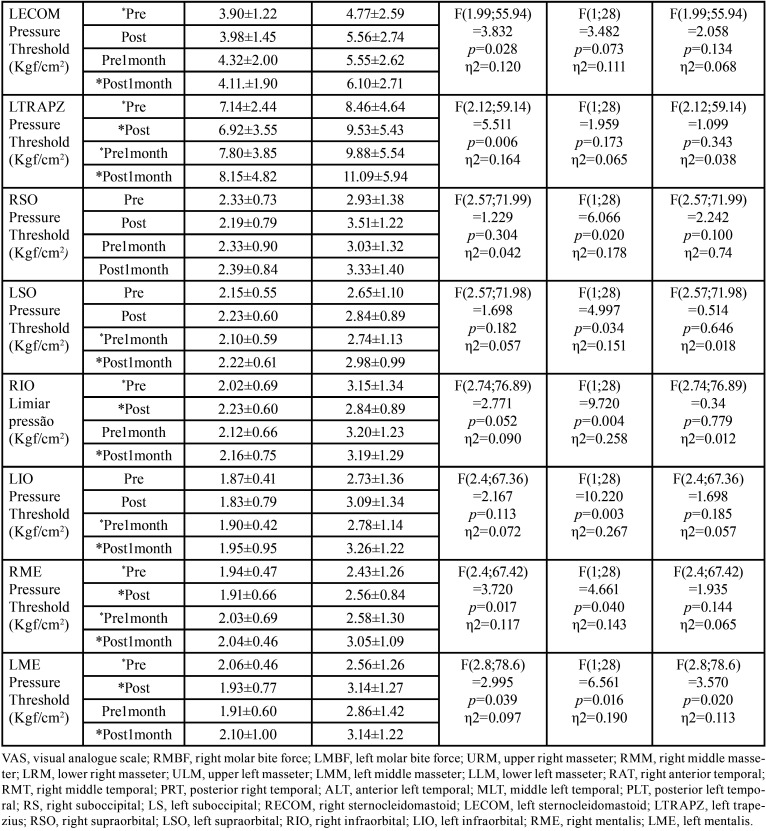
Mean, standard deviation (±), degree of freedom (df), effect size (η2), and p value (< 0.05) of the significant study variables, time, intervention and interaction of Dry Needling (GDN) and Instrumental Myofascial Release (GIMR) techniques.

Table 3 shows the multiple comparisons (Bonferroni test) between the mean time for pain in the head and neck region, strength in the right and left the molar region, and pressure thresholds for the masticatory, facial, and cervical muscles. There was a statistical difference between the time means for the head and neck region, right and left molar region, and most of the muscles evaluated at the pressure threshold. There was also a statistical difference between the means of the interventions for some muscles evaluated at the pressure threshold (Table 4).

**Table 3:**
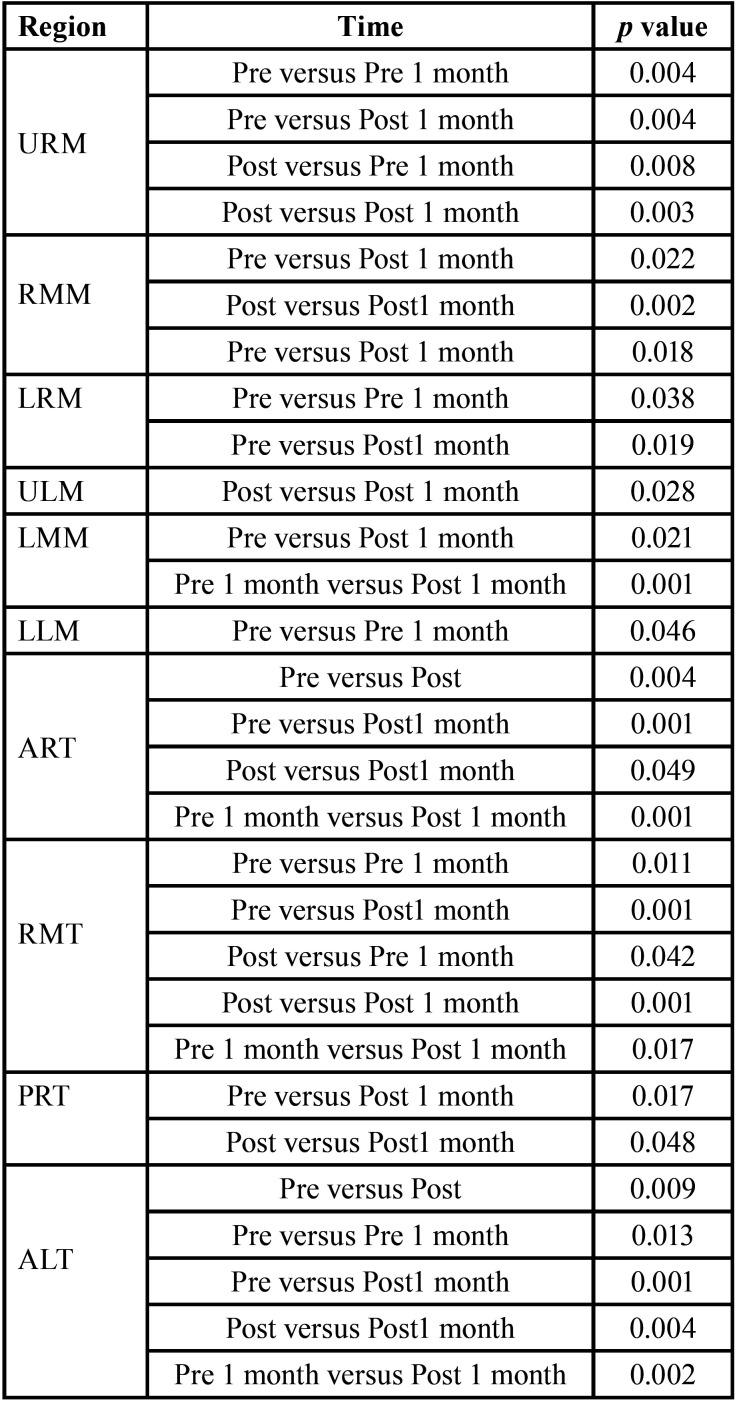
Pairwise comparisons with statistical significance between mean times in the region of masticatory, facial, and cervical muscles at pressure threshold.

**Table 3 cont.**
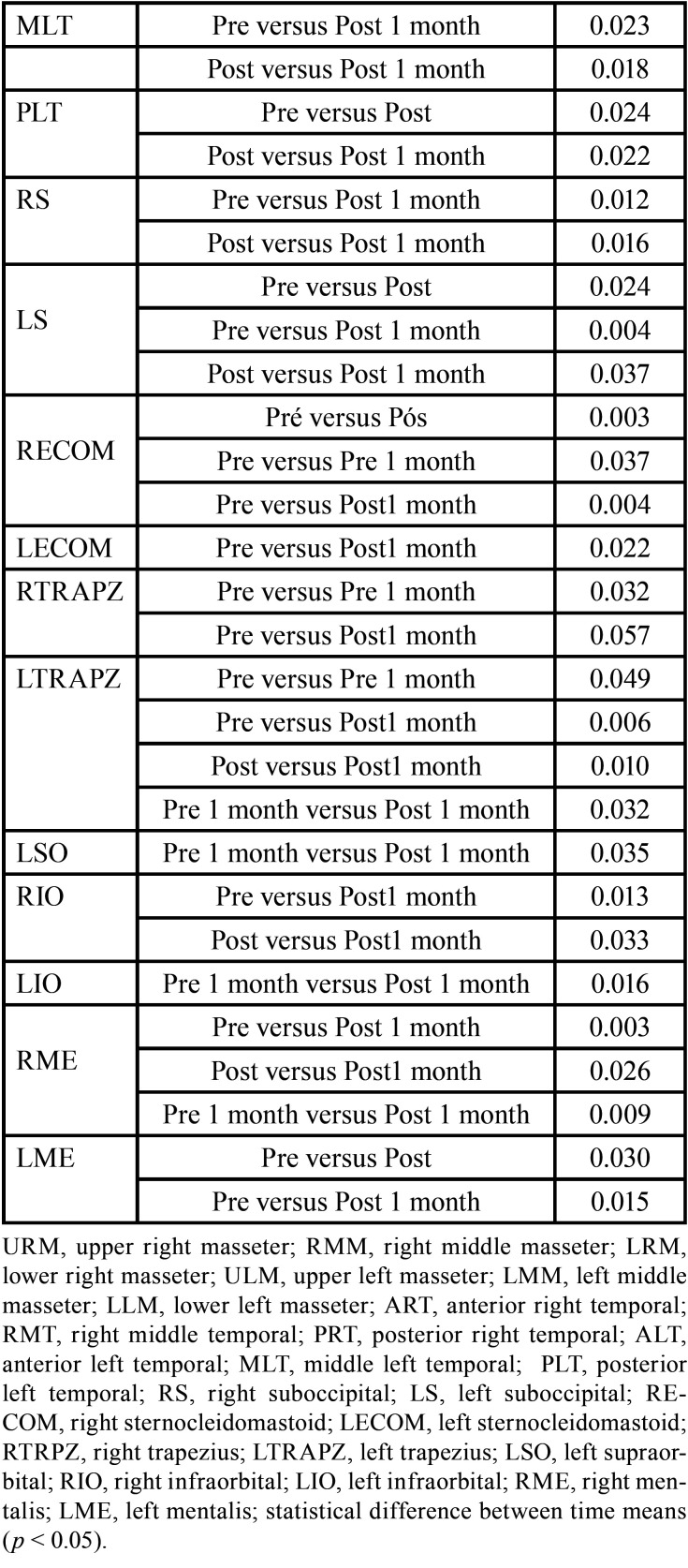
Pairwise comparisons with statistical significance between mean times in the region of masticatory, facial, and cervical muscles at pressure threshold.

**Table 4:**
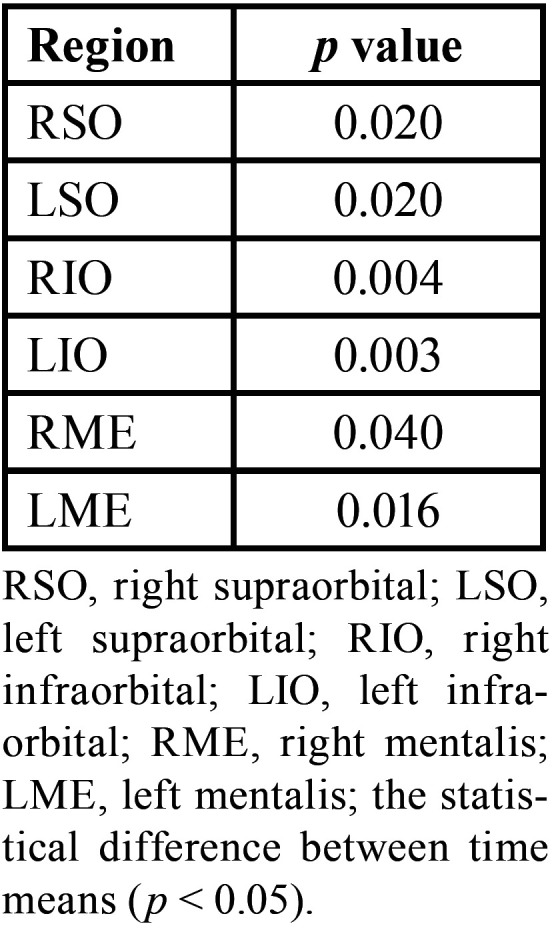
Pairwise comparisons with statistical significance between intervention means in the region of masticatory, facial, and cervical muscles at pressure threshold.

## Discussion

In this study, patients were divided into two groups: GDN and GIMR, which were evaluated pre- and immediately after the first and second interventions. The interval between the two interventions was one month. There was a positive effect on most of the evaluated parameters; therefore, the null hypothesis was rejected.

The pain VAS results showed a reduction in the mean post-intervention pain values for both groups with the effect of time and interaction (time versus intervention) for the head and neck region. The reduction in the mean pain values for the GDN group was gradual until the last assessment. For GIMR, although the reduction in the mean values after the intervention was greater (more than 50% of the initial value), after one month of treatment, the mean value increased and decreased after the second intervention.

The positive effects of both treatments are in line with the literature; however, there is still no consensus on the number of interventions (16). In an attempt to elucidate the effectiveness of these two techniques, explanations have been elaborated, such as the mechanical and neurophysiological effects (17).

From a mechanical standpoint, drilling or massaging the trigger point can disrupt dysfunctional endplates, reduce the overlap between actin and myosin filaments, and decrease acetylcholine levels. Decreased acetylcholine levels may lead to increased muscle blood flow and oxygenation, consequently reducing sarcomere contracture (18).

Regarding the neurophysiological effect, both techniques can reduce peripheral and central sensitization, removing the source of peripheral nociception, modulating spinal dorsal horn activity, and activating the central pain inhibitory pathways (19). Areas with active trigger points exhibit local biochemical changes, including increased availability of pro-inflammatory substances, such as substance P, interleukin 1 beta (IL-1β), and tumor necrosis factor alpha (TNF-a), which activate the muscle nociceptors (20). When the nourishing blood flow is restored, the chemicals that cause pain are removed and cellular repair begins. Repeated treatments can continually interrupt the cycle, prevent regression, and allow long-term repair and recovery (21). This explanation may lead to the results of this study, that is, two interventions were not enough to reduce, but not to completely eliminate, the self-perception of pain, as will be seen by the algometry results.

The results of the maximum molar bite force showed an increase in the mean values after the intervention in both groups, with a statistical effect of time and interaction (time versus intervention) in the region of the right and left molars. An increase in molar bite force indicates that both techniques are effective in treating myofascial pain. However, in the GDN group, after one month of treatment, the mean values decreased, increasing only after the second intervention. In contrast, the mean values of maximal molar bite force in the GIMR group increased continuously and gradually. This difference proves that the results of IMR are long-lasting.

The results of this study revealed a difference between the mean values of teeth 26–36 immediately after the first and second interventions. This result was expected since the absence of a statistical difference between the right and left hemiarch indicated that the patients had a balanced occlusion (22).

The ability to perceive pain in nociceptors showed an effect of time in all the evaluations for the right masseter and only for the middle left masseter. The interaction effect occurred for the right and upper left masseter, indicating that the mean pain tolerance values were higher for the GDN group in the relationship between time and intervention. The increase registered by the right and left masseter muscles was gradual and continuous in the GIMR group. In contrast, in the GDN group, this result was obtained only for the right inferior masseter muscle. For the other evaluations of the right and left masseters (middle, upper, and lower), there was a decrease in pain tolerance immediately after the intervention, with a tendency towards an increase in mean values after one month, when compared to the initial values.

An increase in the tolerance of pain perception in these muscles is desirable, as pain in the masseter muscles is one of the greatest complaints in patients with TMD with the presence of myofascial trigger points (5). As for the decrease in pain tolerance immediately after DN, it is quite common in this technique, generally lasting less than 72 hours after the intervention. This adverse event can promote patient dissatisfaction and reluctance to adhere to the treatment (23).

One of the post-needle-induced pain hypotheses is neuromuscular damage caused by consecutive needle insertions in the muscle, as well as by the hemorrhagic and inflammatory reactions triggered by the needle. A study supported this hypothesis of neuromuscular damage when it identified the lesion caused by the application of DN in the gastrocnemius muscle through magnetic resonance imaging (24).

While the masseter is the most powerful mandibular elevator muscle, the temporalis is considered the mandibular repositioning muscle. The anterior bundles of the temporalis contract during maximum mouth opening. The posterior bundles contract during mandibular retraction and cause contralateral displacement. Thus, the temporal bone is fundamental not only in determining muscle tone in the postural position of the mandible but also in chewing movements (25).

In this study, the effect of time was observed in all the evaluations of the temporal muscles. Continuous gradual increases in the mean values were recorded for the right and middle left temporal regions in both the groups and for the anterior and posterior left temporal regions only in the GIMR group. In the other analyses, there was a decrease in pain tolerance immediately after the intervention, with an increase in the mean values after one month, when compared to the initial values.

These results were similar to those for the masseter muscles, the reasons for which have been discussed earlier. The success of IMR and DN in reducing pain in the temporal and masseter muscles in patients with TMD has been reported in other studies (26).

Regarding the suboccipital muscle, which plays an important role in muscle posture, as it stabilizes the atlanto-occipital joint during head movements, helping to extend, laterally flex, and rotate the neck, an effect of time was observed on this musculature. The interaction effect for the left suboccipital muscle indicated that the mean pain tolerance values were higher for the GDN group in the relationship between time and intervention. Gradual and continuous increases were recorded in the right and left suboccipital regions in the GLMI group, and the left suboccipital regions in the GDN group. For the right suboccipital region in the GDN group, there was a decrease in pain tolerance immediately after the intervention, as already discussed. These results are in agreement with the literature (27).

The sternocleidomastoid muscle is another important cervical muscle that has multiple functions, such as unilateral and bilateral contraction of the cervical region of the spine, which influences the masseter muscle. This study demonstrated that there was an effect of time on the right sternocleidomastoid muscle and an interaction for the left sternocleidomastoid muscle. Given the importance of this muscle, the results are promising as they demonstrate a positive impact of both techniques. These findings corroborate those of other studies that have used these techniques to treat patients with headaches or other disorders (28).

The trapezius muscle is considered the dominant stabilizer of the scapula that contributes not only to the normal mechanics of the scapula but also to the entire shoulder region. Altered activation, poor control, and reduced strength of different parts of the trapezius have been associated with pain and reduced function. Data from this study showed that time had an effect on the left trapezius muscle. The positive impact of both techniques on this muscle is in agreement with the literature (29).

The orbicularis oculi muscle is considered the sphincter of the eyelids and is involved in facial expressions, eye protection, and reflexes. Electrical stimulation of the supraorbital branch of the trigeminal nerve on one side results in ipsilateral short latency and bilateral long-latency orbicularis muscle contraction (30). In contrast to the previous results of this study, this intervention had an effect on the supraorbital and infraorbital muscles. IMR interventions achieved mean values greater than those achieved by DN. The values after the second intervention were higher than those initially recorded.

In contrast, the mentalis muscle is responsible for lifting the lower lip and assisting in facial expression, providing vertical support for the lower lip (31). It was observed that there was an effect of time and intervention on the right and left mentalis muscles, as well as an interaction effect on the left mentalis muscle. The values after the second intervention were higher than those initially recorded. No studies have reported the impact of either technique on the orbicularis oculi or mentalis muscles.

This study had some limitations. Two interventions, one month apart, were not sufficient to eliminate pain. Future research should analyze the impact of treatment with a greater number of interventions and whether these results extend to other populations and clinical situations.

## Conclusions

This study suggests that DN and IMR techniques are viable therapeutic techniques for patients with muscular TMD; however, IMR proved to be more efficient both in the immediate post-intervention evaluation and after one month, as evidenced by the reduction of chronic pain and better performance of the cervico-cranio-mandibular system.
